# Supplementary material for: Chromothripsis during telomere crisis is independent of NHEJ, and consistent with a replicative origin
Source: Genome Res. 2019 May;29(5):737–49. doi: 10.1101/gr.240705.118 (PMC6499312; doi:10.1101/gr.240705.118)
Supplement: Supplemental Material [file supp_gr.240705.118_Supplemental_file_1.zip › contigs/annotated_contigs/DB51/contig.2.DB51_length_544_mean_cov_4.00183823529.docx]

**DB51_length_544_mean_cov_4.00183823529**

```
GTTCCATTACCTCTTTGACCTTCCCTTTCCTCATTCCACTCCTGCATACTGGCCTCCCTGTTCTTCCTGGAAAATACCAGGCACCCTCC
 >chr6:135246738-135247159 - E=7e-233
TCCTTCTTGGCCCTTGCTCTTGTTGCTCACGCTGCCTGGAATGCTCTGCCTTCAGATGCACGCATGGCTCTCTCCTTCCCCTTCGGAGA

GTAGATGTCACCTGAATGAGGGCTTTCCTGCCTACCCTATATACAGTGGTACCTTTTAAAACTTTGACTTCTTTGGAGCTTTATTTTTC

TCCACGGTTCTTACTGTCATCTGACATGCAATATATGTATCCTATCCCTTTACTGACTATCTTCCCTGGACTAGAATGTCAGCTTCTTG

GTCAGTTTTATTCATTGCTGTATCTTTAGACTCTAGAAAGCTCCTGGAACATAGTAGGTGC|ATAG|CCACAGAGAGAATCCTGTTTTA
                                                              >chr20:19432539-19432666
TAACATGTGGACAGACTGAAGCAAGTCAAGGAAAGCAAACGGGATGGGGAGGGAGGCTCTTGGTAAACATTTCCTTCTTTCACTTTATT
- E=9e-65
GGGGCTGAAAGC
```
